# Prevalence of dyslipidemia and associated factors in sedentary occupational population from Shanghai: a cross-sectional study

**DOI:** 10.1186/s13690-024-01245-0

**Published:** 2024-02-08

**Authors:** Dongxing Gu, Dandan Wang, Qinzhong Zhu, Li Luo, Tiantian Zhang

**Affiliations:** 1 Department of Information Center, Huadong Sanatorium, Wuxi, 214065 China; 2 School of Public Health, Fudan University, 130 Dong’an Rd, Xuhui District, Shanghai, 200032 China; 3 Shanghai Institute of Infectious Disease and Biosecurity, Shanghai, 200032 China

**Keywords:** Dyslipidemia, Sedentary occupational population, Prevalence, Risk factor, China

## Abstract

**Background:**

Dyslipidemia is a major risk factor for cardiovascular diseases, but its prevalence and determinants among sedentary occupational population are poorly understood. This study aimed to investigate the prevalence and associated factors for dyslipidemia among a sedentary occupational population in Shanghai, China.

**Methods:**

We collected data from 35,950 sedentary occupational workers on their demographics, social, medical, and family history, lifestyle, anthropometry, and biochemistry. We used the 2016 Chinese guideline for the management of dyslipidemia in adults to define dyslipidemia and its subtypes. We performed multivariate logistic regression to examine the factors associated with dyslipidemia.

**Results:**

The prevalence of dyslipidemia was 29.10%, with 15.86% for high triglycerides (TG), 6.43% for high total cholesterol (TC), 5.37% for high low-density lipoprotein cholesterol (LDL-C), and 14.68% for low high-density lipoprotein cholesterol (HDL-C). Men had a significantly higher prevalence of dyslipidemia than women (39.64% vs. 12.43%, *P* < 0.01). Factors associated with dyslipidemia included older age, being married, longer sedentary time while resting, frequent intake of animal viscera, current smoking, hypertension, diabetes, and obesity. Current drinking was associated with a 1.24 times higher prevalence of high TG (*P* < 0.01). Current smokers were less likely to have low HDL-C than non-smokers.

**Conclusions:**

Our present study, in a population of 35,950 sedentary occupational workers from Shanghai, demonstrated a prevalence of dyslipidemia, but lower than in other previous studies without the limitation of occupational characteristics. Prevention and control measures for dyslipidemia should take into account the characteristics and related factors for this population group.

**Table:** 

Text box 1. Contributions to the literature
• There is limited evidence on the prevalence of dyslipidemia and associated factors among sedentary occupational population.
• Current smoking, hypertension, diabetes, and obesity are associated with both high TG and low HDL-C. Animal viscera and adequate vegetable intake are associated with high TC and high LDL-C.
• Effective strategies targeting associated factors among sedentary occupational population are needed.

## Background

Dyslipidemia is a disorder of lipid metabolism that results in abnormal blood levels of lipids, such as total cholesterol (TC), low-density lipoprotein cholesterol (LDL-C), triglycerides (TG), and high-density lipoprotein cholesterol (HDL-C) [1]. Dyslipidemia is a major risk factor for cardiovascular disease (CVD), the leading cause of death and disability worldwide accounting for 32% in 2019, by causing atherosclerosis, inflammation, and thrombosis [2–4]. Dyslipidemia is becoming more prevalent globally, especially in developing countries and among the elderly [5]. Based on national health survey data, the prevalence of dyslipidemia was 53% in America and continued to grow in Japan, especially in the elderly [5, 6]. An analysis of 134,074 Canadian adults showed that 35.8% had at least one abnormal lipid component [7]. On the basis of cardiovascular health measures of the household population in 2017, Canadian adults (aged 20–79 years) had a mean TC level of 3.7 mmol/L [8]. The national survey in 2012 showed that the mean TC level of Chinese adults was 4.5 mmol/L, and the prevalence of dyslipidemia in adults was 40.4% [9]. In 2021, the prevalence of dyslipidemia among the elderly in China was 47.0% [10]. Without timely and effective intervention, the burden of dyslipidemia and its complications will continue to grow. However, many Chinese adults have low awareness and control of dyslipidemia, which poses a great challenge for CVD prevention in China [5, 11]. Therefore, it is important to identify the potential factors influencing dyslipidemia and to implement targeted management strategies from a public health perspective.

Many studies have found that sedentary behavior is a risk factor for chronic diseases, such as obesity and metabolic syndrome, and in particular increases the risk of dyslipidemia [12–14]. Adults usually spend approximately 1/2 of their weekday awake time working, and the pattern of sedentary behavior varies by occupation [15]. In urban areas, occupational population with sedentary work patterns mainly come from three groups: government-related units, social service agencies (including educational institutions, public health institutions, social welfare institutions, etc.) and enterprises. These groups have a common feature: high mental work and less physical activity. However, they may face different challenges and opportunities for dyslipidemia prevention and control than other occupational groups. For example, they may have more access to health education and screening services, but also more exposure to stress and sedentary working pattern. It is difficult to change the pattern or activity in the workplace, so understanding their burden of dyslipidemia and related factors from a public health management perspective is an effective path to prevent and manage dyslipidemia among sedentary occupational population. Previous epidemiological studies have mainly examined the regional and gender differences of dyslipidemia, as well as its prevalence in the elderly population [16–19]. There is a lack of research to determine the burden of dyslipidemia among sedentary occupational population in the last few years, and related factors of dyslipidemia in sedentary occupational population are rather limited and outdated. Therefore, this study aims to investigate the prevalence and types of dyslipidemia among sedentary occupational population in Shanghai using a cross-sectional survey design. The study also aims to identify the potential factors that influence dyslipidemia among this occupational group using multivariate logistic regression analysis. The findings of this study may help to provide evidence-based recommendations for preventing and controlling dyslipidemia among sedentary occupational population in China.

## Methods

### Study population

Study participants were recruited from 226,296 people who underwent a health checkup in Huadong Sanatorium from January 2019 to December 2021. This hospital is a medical institution in Shanghai taking health checkups as its main business and serving for the occupational population, from enterprises, social service agencies, and some government-related units. The agency set up a routine health questionnaire in 2019–2021 as part of the agency’s “book a checkup – fill out a questionnaire – conduct a checkup” process. This practice aims to collect information on the demographic and social characteristics, medical history and family history, and lifestyle factors, so that doctors can provide more precise diagnoses and follow-up health management based on the results of checkup. People booked online fill in the online questionnaire mainly. People booked offline or booked online without completing the questionnaire will be instructed to fill out the questionnaire by nurses before or doctors during the health checkup. All people were signed with electronic informed consent before filling in.

Study subjects were selected using a two-stage cluster sampling. In the first phase, based on the nature of institution, the physical examination groups were divided into enterprise workers, social service agencies workers, and government-related units workers. A total of 5,538 institutions were involved. In the second phase, we randomly sampled 1,108 institutions at 20% from institutions of different nature. A total of 52,924 people were involved. After excluding those who were pregnant, lactating, or had severe mental or physical illnesses, and whose questionnaire filling time was more than 6 months apart from the health checkup time, 42,259 people were included in this sample. Excluding people whose information was incomplete or inconsistent, the final sample size was 35,050 participants. The study was approved by the Ethics and Research Committee of Huadong Sanatorium Health Examination Center (approval number: ECHS2023-07).

### Data collection

We conducted a cross-sectional survey to examine the prevalence and related factors of dyslipidemia among sedentary occupational population. The questionnaire was designed to gather information on demographic and social characteristics (including age, sex, attribute of work organization, marital status, educational level), medical history and family history (including hypertension, diabetes, coronary heart disease (CHD)), and lifestyle factors (including regular diet, nighttime diet, vegetable intake, meat intake, fat intake, animal viscera intake, sugary beverages intake, sedentariness time at rest, smoking status, drinking status, physical activity, average sleep times).

For demographic and social characteristics, the attributes of work organization were divided into social service agencies, enterprises, and agency units. We classified the educational attainment as primary school or below, junior school, middle school, bachelor’s degree, master degree or above, and the marital status as married, divorced or widowed, and single.

For health behaviors, nighttime diet was classified into never, occasional (< 1 time per week), and frequent (≥ 1 time per week) [20, 21]. Based on 2022 Dietary Guidelines for Chinese Residents, vegetable intake was categorized as poor (< 300 gram per day), adequate (≥ 300 gram per day) and meat intake was categorized as poor (< 120 gram per day), adequate (120–250 gram per day), excessive (> 250 gram per day) [22]. Fat intake and animal viscera intake were classified into one of the following categories: never, occasional (1–2 times per week), frequent (≥ 3 times per week). Sugary beverage intake was divided into never or occasional (1–2 times per week), frequent (3–5 times per week), and every day (> 5 times per week) [23]. Sedentariness was defined as continuous sitting for more than 2 h based on the Canadian Sedentary Behavior Guidelines [24]. Former drinking and former smoking were defined as participants who didn’t drink or smoke during the past 12 months [25]. Physical activity was classified according to the number and time of aerobic exercise per week as insufficient (< 3 times per week with sessions shorter than 30 min), adequate (> 3 times per week with sessions longer than 30 min), general (< 3 times per week with sessions longer than 30 min, or > 3 times per week with sessions shorter than 30 min) [26]. This part of the data was obtained by participants’ recall.

### Physical examinations

Physical examinations were administered in the morning after overnight fasting by professional nurses who received standardized training. Two consecutive blood pressure measurements were taken at a 45-min interval using a mercury sphygmomanometer with an appropriate cuff size. The mean blood pressure value of the two readings was used [27, 28]. Serum TC, TG, LDL-C, HDL-C, and fasting blood glucose (FBG) were measured by an automatic analyzer with a fasting blood sample using enzymatic methods. The coefficients of variation for all biochemical assays were less than 5%. Weight and height were measured with the participants wearing light weight clothes and no shoes. Body mass index (BMI) was calculated by dividing body weight in kilograms by height in meters squared. The waist circumference (WC) was horizontally measured at the level of their navel using a nonstretchable tape.

### Definitions

According to the 2016 Chinese Guidelines for Prevention and Treatment of Dyslipidemia in Adults [29], dyslipidemia was defined as the presence of any of these four indicators: TC ≥ 6.22 mmol/L, TG ≥ 2.26 mmol/L, LDL-C ≥ 4.14 mmol/L, HDL-C < 1.04 mmol/L. Hypertension was considered with systolic blood pressure (SBP) ≥ 140 mmHg or diastolic blood pressure (DBP) ≥ 90 mmHg or having been diagnosed by doctors according to the 2018 Chinese Guidelines for Prevention and Treatment of Hypertension [30]. Diabetes was considered with FPG ≥ 7.0 mmol/L or having been diagnosed by doctors according to the Chinese Guidelines for Prevention and Control of type 2 diabetes [31]. BMI was calculated as weight in kilograms divided by height in meters squared. Participants with BMI ≥ 28.0 kg/m2 were diagnosed with obesity according to the Guidelines for Prevention and Control of Overweight and Obesity in Chinese Adults [32–34].

### Statistical analysis

We used descriptive and inferential statistics to analyze the data. Descriptive statistics were used to summarize the characteristics of the study population and compare the differences between groups. Continuous variables were expressed as mean ± standard deviation (SD) and categorical variables as frequencies and percentages. The unpaired Student’s *t*-test or Wilcoxon rank sum test, and Pearson’s chi-square test were used to compare differences between groups. Inferential statistics were used to identify the risk factors for dyslipidemia and its subtypes using multiple logistic regression models. We used a stepwise selection procedure with an entry criterion of *p* < 0.05 and a removal criterion of *p* > 0.10 to select the variables for the final models. The association between dyslipidemia and risk factors was assessed by adjusted odds ratios (ORs) and 95% confidence intervals (CIs). All statistical analyses were carried out using STATA version 13.0 (Stata Corp LP., College Station, TX, USA). A two-sided p-value of less than 0.05 was considered statistically significant.

## Results

### Demographic characteristics

A total of 35,950 participants were included in this study, including 22,017 males (61.24%) and 13,933 females (38.76%). The mean age of the participants was 45.13 (SD = 10.31) years, ranging from 20 to 89 years. Participants from social service agencies, enterprises, and agency units accounted for 23.43%, 38.64%, and 37.93%, respectively. Of the 35,950 participants in this study, 34,439 (93.02%) had a bachelor’s degree or above, 20.59% smoked, 45.87% drank alcohol, 44.04% had insufficient physical activity, 72.23% had sedentary behavior, and 13.30% had obesity. The mean levels of TC, TG, HDL-C and LDL-C were 4.83 ± 0.89 mmol/L, 1.51 ± 1.25 mmol/L, 1.36 ± 0.33 mmol/L, and 2.97 ± 0.70 mmol/L, respectively. The demographic characteristics among sedentary occupational population is shown in Table 1 (see Additional file 1: Table 1).

**Table 1:** Prevalence of dyslipidemia by subpopulation

Variables	Total (*n*=35,950)	Dyslipidemia (*n*=10,460)	Non-dyslipidemia (*n*=25,490)	t/X^2^	P-value
**Age, mean (SD)**	45.13(10.31)	47.55(9.66)	44.17(10.40)	28.87	<0.001
**Gender, n (%)**				3063.63	<0.001
Men	22,017(61.24)	8728(39.64)	13,289(60.36)		
Women	13,933(38.76)	1732(12.43)	12,201(87.57)		
**Age group, n (%)**				830.06	<0.001
20-29	2466(6.86)	330(13.38)	2136(86.62)		
30-39	9028(25.11)	2025(22.43)	7003(77.57)		
40-49	11,300(31.43)	3326(29.43)	7974(70.57)		
50-59	10,154(28.24)	3662(36.06)	6492(63.94)		
60-69	2777(7.72)	1048(37.74)	1729(62.26)		
≥70	225(0.63)	69(30.67)	156(69.33)		
**Marital status, n (%)**				325.69	<0.001
Single	4411(12.27)	774(17.55)	3637(82.45)		
Divorced or widowed	851(2.37)	251(29.49)	600(70.51)		
Married	30,688(85.36)	9435(30.74)	21,253(69.26)		
**Educational level, n (%)**				147.65	<0.001
Primary school or below	140(0.39)	47(33.57)	93(66.43)		
Junior school	600(1.67)	234(39.00)	366(61.00)		
Middle school	1771(4.93)	639(36.08)	1132(63.92)		
Bachelor degree	22,728(65.11)	6810(29.96)	15,918(70.04)		
Master degree or above	11,711(29.79)	2730(24.49)	7981(74.51)		
**Work organization, n (%)**				71.6	<0.001
Social service agencies	8423(23.43)	2279(27.06)	6144(72.94)		
Enterprises	13,890(38.64)	4393(31.63)	9497(68.37)		
Agency units	13,637(37.93)	3788(27.78)	9849(72.22)		
**Dietary habit, n (%)**				3.26	0.196
Poor	387(1.08)	124(32.04)	263(67.96)		
Intermediate	5572(15.50)	1659(29.77)	3913(70.23)		
Ideal	29,991(83.42)	8677(28.93)	21,314(71.07)		
**Nighttime diet, n (%)**				18.86	<0.001
Never	19,916(55.40)	5619(28.21)	14,297(71.79)		
Occasional	15,369(42.75)	4624(30.09)	10,745(69.91)		
Frequent	665(1.85)	217(32.63)	448(67.37)		
**Vegetable intake, n (%)**				88.97	<0.001
Poor	25,670(71.40)	7836(30.53)	17,834(69.47)		
Adequate	10,280(28.60)	2624(25.53)	7656(74.47)		
**Meat intake, n (%)**				4.87	0.087
Poor	25,647(71.34)	7405(28.87)	18,242(71.13)		
Adequate	8452(23.51)	2477(29.31)	5975(70.69)		
Excessive	1851(5.15)	578(31.23)	1273(68.77)		
**Animal viscera intake, n (%)**				29.19	<0.001
Never	10,345(28.78)	2880(27.84)	7465(72.16)		
Occasional	24,617(68.48)	7228(29.36)	17,389(70.64)		
Frequent	988(2.75)	352(35.63)	636(64.37)		
**Sugary beverages intake, n (%)**				2.59	0.274
Never or occasional	31,567(87.81)	9150(28.99)	22,417(71.01)		
Frequent	3858(10.73)	1143(29.63)	2715(70.37)		
Everyday	525(1.46)	167(31.81)	358(68.19)		
**Sedentariness in rest, n (%)**				15.08	0.002
<2 h	9982(27.77)	2826(28.31)	7156(71.69)		
2～4 h	15,271(42.48)	4505(29.50)	10,766(70.50)		
4～6 h	6246(17.37)	1903(30.47)	4343(69.53)		
>6 h	4451(12.38)	1226(27.54)	3225(72.46)		
**Sleep time, n (%)**				86.78	<0.001
<5 h	1995(5.55)	671(33.63)	1324(66.37)		
5～7 h	25,317(70.42)	7591(29.98)	17,726(70.02)		
7～9 h	8476(23.58)	2150(25.37)	6326(74.63)		
>9 h	162(0.45)	48(29.63)	114(70.37)		
**Physical activity, n (%)**				12.11	0.002
Insufficient	15,833(44.04)	4509(28.48)	11,324(71.52)		
General	9083(25.27)	2771(30.51)	6312(69.49)		
Sufficient	11,034(30.69)	3180(28.82)	7854(71.18)		
**Smoking status, n (%)**				2017.88	<0.001
Never smoker	26,915(74.87)	6176(22.95)	20,739(77.05)		
Ever smoker	1632(4.54)	651(39.89)	981(60.11)		
Current smoker	7403(20.59)	3633(49.07)	3770(50.93)		
**Drinking status, n (%)**				1414.41	<0.001
Never drinker	19,053(53.00)	3929(20.62)	15,124(79.38)		
Ever drinker	408(1.13)	174(42.65)	234(57.35)		
Current drinker	16,489(45.87)	6357(38.55)	10,132(61.45)		
**CHD family history, n (%)**				2.87	<0.001
Yes	1488(4.14)	462(31.05)	1026(68.95)		
No	34,462(95.86)	9998(29.01)	24,464(70.99)		
**CHD, n (%)**				8.58	0.003
Yes	212(0.59)	81(38.21)	131(61.79)		
No	35,738(99.41)	10,379(29.04)	25,359(70.96)		
**Hypertension, n (%)**				1209.27	<0.001
Yes	5996(16.68)	2861(47.72)	3135(52.28)		
No	29,954(83.32)	7599(25.37)	22,355(74.63)		
**Diebetes, n (%)**				760.23	<0.001
Yes	2045(5.69)	2861(47.72)	3135(52.28)		
No	33,905(94.31)	7599(25.37)	22,355(74.63)		
**Obesity, n (%)**				1602.2	<0.001
Yes	4782(13.30)	2562(53.58)	2220(46.42)		
No	31,168(86.70)	7898(25.34)	23,270(74.66)		
**BMI, mean±SD**	24.34(3.42)	26.09(3.28)	23.63(3.22)	65.68	<0.001
**WC, mean±SD**	81.79(12.61)	87.47(12.08)	79.45(12.08)	57.19	<0.001
**TC, mean (SD)**	4.83(0.89)	5.21(1.15)	4.67(0.70)	54.67	<0.001
**TG, mean (SD)**	1.51(1.25)	2.61(1.77)	1.05(0.45)	130.62	<0.001
**HDL-C, mean (SD)**	1.36(0.33)	1.13(0.31)	1.45(0.29)	91.42	<0.001
**LDL-C, mean (SD)**	2.97(0.70)	3.30(0.86)	2.83(0.57)	58.21	<0.001

### Prevalence of dyslipidemia

The prevalence of dyslipidemia was 29.10%, with high TG (15.86%) being the most common component, followed by low HDL-C (14.68%), high TC (6.43%) and high LDL-C (5.37%). The prevalence of dyslipidemia and its components among sedentary occupational population is shown in Tables 1 and 2, respectively (see Additional file 1: Table 1; see Additional file 2: Table 2). The prevalence of dyslipidemia was significantly higher in men than in women (39.64% vs. 12.43%, *P* < 0.01). The prevalence of dyslipidemia increased with increasing age, but decreased in the ≥ 70 years age group (*P* < 0.01). Marital status, education level, nighttime diet, animal viscera intake, sedentariness in rest, sleep time, physical activity, smoking status and drinking status, chronic diseases, family history, and BMI were also associated with the prevalence of dyslipidemia (*P* < 0.01 for all). Sedentary occupational population who had insufficient vegetable intake, diabetes, hypertension, CHD, CHD family history or obesity had a higher prevalence of dyslipidemia (*P* < 0.01). The prevalence of all dyslipidemia components varied by gender, age, education level, work organization, dietary habits, sedentary behavior, sleep time, physical activity, smoking status, drinking status, chronic diseases, family history, and BMI (*P* < 0.01 for all). The prevalence of high TG and low HDL-C was higher in participants who often ate late at night (*P* < 0.01 for both). The prevalence of high TG and low HDL-C was also higher in participants who had insufficient physical activity compared with those who had sufficient physical activity (*P* < 0.05 for both).

**Table 2:** Prevalence of dyslipidemia components by subpopulation

Variables	High TG (*n*=5703)	High TC (*n*=2311)	High LDL-C (*n*=1932)	Low HDL-C (*n*=5278)
**Gender, n (%)**				
Men	5110(23.27)	1489(6.78)	1310(6.24)	4772(22.72)
Women	593(4.28)	822(5.94)	622(4.71)	506(3.83)
*P*-value	<0.001	0.001	<0.001	<0.001
**Age group, n (%)**				
20-29	99(4.06)	73(2.99)	86(3.67)	183(7.81)
30-39	993(11.06)	378(4.21)	351(4.15)	1169(13.83)
40-49	1942(17.24)	690(6.12)	569(5.27)	171(15.91)
50-59	2087(20.61)	917(9.05)	728(7.48)	1654(17.00)
60-69	551(19.90)	240(8.67)	187(7.01)	517(19.38)
≥70	31(13.84)	13(5.80)	11(5.14)	38(17.76)
*P*-value	<0.001	<0.001	<0.001	<0.001
**Marital status, n (%)**				
Single	320(7.32)	197(4.51)	188(4.47)	404(9.60)
Divorced or widowed	124(14.61)	75(8.83)	65(8.06)	110(13.65)
Married	5259(17.19)	2039(6.67)	1679(5.75)	4764(16.32)
*P*-value	<0.001	<0.001	<0.001	<0.001
**Educational level, n (%)**				
Primary school or below	27(19.42)	7(5.04)	3(2.34)	26(20.31)
Junior school	147(24.50)	52(8.67)	42(7.49)	112(19.96)
Middle school	385(21.81)	129(7.31)	89(5.40)	321(19.49)
Bachelor degree	3796(16.76)	1547(6.83)	1289(5.99)	3376(15.70)
Master degree or above	1348(12.65)	576(5.41)	509(4.91)	1443(13.93)
*P*-value	<0.001	<0.001	<0.001	<0.001
**Work organization, n (%)**				
Social service agencies	1141(13.69)	507(6.08)	435(5.62)	1175(15.19)
Enterprises	2521(18.18)	946(6.82)	796(5.96)	2208(16.52)
Agency units	2041(15.00)	858(6.31)	701(5.35)	1895(14.46)
*P*-value	<0.001	0.063	0.102	<0.001
**Nighttime diet, n (%)**				
Never	2926(14.75)	1287(6.49)	1079(5.68)	2827(14.87)
Occasional	2650(17.32)	979(6.40)	813(5.58)	2333(16.02)
Frequent	127(19.16)	45(6.79)	40(6.31)	118(18.61)
*P*-value	<0.001	0.886	0.719	0.001
**Vegetable intake, n (%)**				
Poor	4380(17.13)	1706(6.67)	1430(5.85)	3952(16.18)
Adequate	1323(12.93)	605(5.91)	502(5.14)	1326(13.57)
*P*-value	<0.001	0.008	0.009	<0.001
**Animal viscera intake, n (%)**				
Never	1426(13.83)	710(6.89)	607(6.15)	1486(15.06)
Occasional	4080(16.64)	1516(6.18)	1248(5.34)	3624(15.50)
Frequent	197(20.00)	85(8.63)	77(8.11)	168(17.68)
*P*-value	<0.001	0.001	<0.001	0.091
**Sedentariness in rest, n (%)**				
<2 h	1554(15.63)	597(6.00)	468(4.90)	1437(15.04)
2～4 h	2464(16.20)	1006(6.61)	847(5.84)	2229(15.37)
4～6 h	1029(16.55)	426(6.85)	371(6.28)	988(16.73)
>6 h	656(14.49)	282(6.36)	246(5.80)	624(14.70)
*P*-value	0.056	0.129	<0.001	0.014
**Sleep time, n (%)**				
<5 h	370(18.63)	158(7.96)	124(6.56)	323(17.09)
5～7 h	4125(16.36)	1695(6.72)	1432(5.93)	3812(15.78)
7～9 h	1186(14.05)	449(5.32)	369(4.61)	1115(13.93)
>9 h	22(13.58)	9(5.56)	7(4.64)	28(18.54)
*P*-value	<0.001	<0.001	<0.001	<0.001
**Physical activity, n (%)**				
Insufficient	2466(15.65)	1008(6.40)	845(5.62)	2309(15.35)
General	1552(17.16)	562(6.21)	484(5.59)	1411(16.31)
Sufficient	1685(15.32)	741(6.74)	603(5.74)	1558(14.82)
*P*-value	0.001	0.299	0.896	0.016
**Smoking status, n (%)**				
Never smoker	2955(11.03)	1586(5.92)	1355(5.29)	2981(11.65)
Ever smoker	382(23.46)	122(7.49)	90(5.78)	322(20.67)
Current smoker	2366(32.02)	603(8.16)	487(6.91)	1975(28.01)
*P*-value	<0.001	<0.001	<0.001	<0.001
**Drinking status, n (%)**				
Never drinker	1676(8.84)	1114(5.88)	947(5.24)	1971(10.91)
Ever drinker	91(22.30)	14(3.43)	15(3.83)	111(28.32)
Current drinker	3936(23.93)	1183(7.19)	970(6.16)	3196(20.30)
*P*-value	<0.001	<0.001	<0.001	<0.001
**CHD family history, n (%)**				
Yes	251(16.95)	117(7.90)	98(6.79)	234(16.22)
No	5452(15.88)	2194(6.39)	1834(5.60)	5044(15.40)
*P*-value	0.273	0.021	0.055	0.399
**CHD, n (%)**				
Yes	36(17.06)	6(2.84)	7(3.37)	57(27.40)
No	5667(15.92)	2305(6.48)	1925(5.66)	5221(15.36)
*P*-value	0.652	0.032	0.153	<0.001
**Hypertension, n (%)**				
Yes	1879(31.41)	505(8.44)	389(6.81)	1475(25.83)
No	3824(12.82)	1806(6.06)	1543(5.42)	3803(13.35)
*P*-value	<0.001	<0.001	<0.001	<0.001
**Diebetes, n (%)**				
Yes	758(37.10)	217(10.62)	166(8.45)	642(32.69)
No	4945(14.65)	2094(6.20)	1766(5.48)	4636(14.38)
*P*-value	<0.001	<0.001	<0.001	<0.001
**Obesity, n (%)**				
Yes	1633(34.21)	398(8.34)	354(7.72)	1499(32.69)
No	4070(13.12)	1913(6.16)	1578(5.33)	3779(12.76)
*P*-value	<0.001	<0.001	<0.001	<0.001

### Factors influencing dyslipidemia

To identify the main factors associated with dyslipidemia, we performed multivariate stepwise logistic regression analyses with dyslipidemia and its components (high TC, high TG, high LDL-C, and low HDL-C) as the dependent variable and the demographic and lifestyle variables as the independent variables. The results are presented in Table 3 (see Additional file 3: Table 3). The factors that were associated with higher odds of dyslipidemia were male gender (OR = 3.20, *P* < 0.01), older age, being married or having a partner, longer sedentary time, frequent animal viscera consumption (OR = 1.17, *P* < 0.05), current smoking (OR = 1.54, *P* < 0.01), having hypertension (OR = 1.43, *P* < 0.01), diabetes (OR = 1.81, *P* < 0.01), or obesity (OR = 2.33, *P* < 0.01). On the other hand, the factor that was associated with lower odds of dyslipidemia was adequate vegetable intake (OR = 0.88, *P* < 0.01) and sufficient physical activity (OR = 0.81, *P* < 0.01). We also found that some factors had different effects on different subtypes of dyslipidemia. Although no effect of drinking status on dyslipidemia was observed, participants with current drinking had a 1.24 fold increased prevalence of high TG (*P* < 0.01). Male gender, being married or having a partner, and occasional animal viscera consumption increased the risk of high TG but decreased the risk of high TC. Older age (40–69 years), frequent animal viscera consumption, sedentariness for 2–6 h, diabetes, and obesity increased the risk of both high TC and high LDL-C. Current smoking, hypertension, diabetes, and obesity increased the risk of both high TG and low HDL-C. Nighttime diet increased the risk of high TG, but not low HDL-C. Current smokers were less likely to have low HDL-C than non-smokers.

**Table 3:** Multivariate stepwise logistic regression analysis of factors associated with dyslipidemia

Variables	High TG		High TC		High LDL-C		Low HDL-C		Dyslipidemia	
	Adjusted OR (95%CI)	P	Adjusted OR (95%CI)	P	Adjusted OR (95%CI)	P	Adjusted OR (95%CI)	P	Adjusted OR (95%CI)	P
**Gender**										
Women	1		1		1		1		1	
Men	3.70(3.34, 4.11)	<0.001	0.88(0.78, 0.99)	0.05	1.25(1.10, 1.42)	0.001	6.31(5.67, 7.03)	<0.001	3.20(2.98, 3.43)	<0.001
**Age group**										
20-29	1		1		1		1		1	
30-39	2.22(1.76, 2.80)	<0.001	1.59(1.21, 2.10)	0	1.21(0.93, 1.58)	0.158	1.39(1.15, 1.68)	0.001	1.50(1.29, 1.73)	<0.001
40-49	3.09(2.44, 3.92)	<0.001	2.45(1.84, 3.26)	<0.001	1.61(1.22, 2.13)	0.001	1.36(1.12, 1.66)	0.002	1.90(1.63, 2.21)	<0.001
50-59	3.08(2.42, 3.92)	<0.001	3.80(2.84, 5.08)	<0.001	2.42(1.82, 3.21)	<0.001	1.18(0.96, 1.44)	0.112	2.19(1.87, 2.56)	<0.001
60-69	2.41(1.86, 3.12)	<0.001	3.71(2.70, 5.10)	<0.001	2.31(1.68, 3.18)	<0.001	1.26(0.90, 1.41)	0.298	1.94(1.63, 2.31)	<0.001
≥70	1.71(1.08, 2.72)	0.02	2.44(1.30, 4.60)	0.01	1.69(0.86, 3.31)	0.127	1.05(0.69, 1.60)	0.805	1.47(1.05, 2.06)	0.03
**Marital status**										
Single	1		1		1		1		1	
Divorced or widowed	1.11(0.87, 1.43)	0.4	1.05(0.78, 1.41)	0.77	1.24(0.90, 1.70)	0.19	1.25(0.97, 1.61)	0.088	1.26(1.04, 1.53)	0.02
Married	1.20(1.04, 1.38)	0.01	0.84(0.70, 1.01)	0.06	0.89(0.74, 1.08)	0.253	1.29(1.13, 1.48)	<0.001	1.17(1.05, 1.29)	0
**Educational level**										
Primary school or below	1		1		1		1		1	
Junior school	1.14(0.69, 1.87)	0.61	1.63(0.72, 3.69)	0.24	3.22(0.98, 10.60)	0.054	0.86(0.52, 1.43)	0.554	1.11(0.73, 1.69)	0.64
Middle school	1.03(0.64, 1.65)	0.9	1.36(0.62, 2.98)	0.45	3.23(0.69, 7.18)	0.178	0.85(0.53, 1.36)	0.49	0.98(0.66, 1.46)	0.94
Bachelor degree	0.99(0.63, 1.56)	0.97	1.52(0.71, 3.27)	0.28	2.93(0.93, 9.26)	0.066	0.81(0.51, 1.29)	0.374	0.99(0.68, 1.46)	0.98
Master degree or above	0.86(0.55, 1.36)	0.53	1.37(0.63, 2.96)	0.43	2.66(0.84, 8.42)	0.096	0.82(0.52, 1.31)	0.4	0.96(0.65, 1.40)	0.82
**Work organization**										
Social service agencies	1		1		1		1		1	
Enterprises	1.15(1.05, 1.24)	0	1.07(1.96, 1.20)	0.24	1.03(0.91, 1.17)	0.619	0.96(0.88, 1.04)	0.32	1.07(1.01, 1.15)	0.03
Agency units	1.01(0.94, 1.11)	0.69	1.02(0.91, 1.14)	0.74	0.93(0.82, 1.05)	0.236	0.87(0.81, 0.96)	0.005	0.96(0.90, 1.03)	0.29
**Nighttime diet**										
Never	1		1		1		1		1	
Occasional	1.14(1.06, 1.22)	<0.001	1.16(1.06, 1.27)	0	1.08(0.98, 1.20)	0.118	0.97(0.90, 1.04)	0.367	1.08(1.02, 1.14)	0.01
Frequent	1.26(1.01, 1.57)	0.04	1.27(0.93, 1.75)	0.13	1.21(0.86, 1.69)	0.275	1.04(0.83, 1.29)	0.759	1.16(0.97, 1.40)	0.11
**Vegetable intake**										
Poor	1		1		1		1		1	
Adequate	0.86(0.80, 0.92)	<0.001	0.88(0.80, 0.97)	0.01	0.88(0.79, 0.98)	0.022	0.95(0.88, 1.02)	0.174	0.88(0.84, 0.94)	<0.001
**Animal viscera intake**										
Never			1		1		1		1	
Occasional	1.09(1.01, 1.17)	0.02	0.89(0.81, 0.98)	0.02	0.86(0.76, 0.95)	0.004	0.94(0.87, 1.00)	0.064	0.99(0.93, 1.04)	0.6
Frequent	1.12(0.97, 1.40)	0.1	1.33(1.04, 1.69)	0.02	1.34(1.04, 1.73)	0.023	0.89(0.74, 1.08)	0.24	1.17(1.01, 1.36)	0.05
**Sedentariness in rest**										
<2 h	1		1		1		1		1	
2～4 h	1.08(0.99, 1.16)	0.06	1.16(1.04, 1.29)	0.01	1.24(1.10, 1.39)	<0.001	1.03(0.96, 1.11)	0.41	1.10(1.04, 1.17)	0
4～6 h	1.06(0.97, 1.16)	0.22	1.21(1.06, 1.38)	0	1.34(1.16, 1.55)	<0.001	1.11(1.01, 1.22)	0.035	1.12(1.04, 1.21)	0
>6 h	1.11(1.01, 1.24)	0.05	1.16(0.99, 1.35)	0.05	1.27(1.08, 1.49)	0.004	1.09(0.98, 1.22)	0.114	1.12(1.03, 1.22)	0.01
**Sleep time**										
<5 h	1		1		1		1		1	
5～7 h	1.04(0.91, 1.18)	0.59	0.98(0.82, 1.16)	0.81	1.01(0.84, 1.23)	0.899	1.01(0.88, 1.15)	0.918	0.99(0.89, 1.10)	0.88
7～9 h	1.08(0.94, 1.25)	0.26	0.86(0.71, 1.05)	0.13	0.86(0.69, 1.06)	0.157	1.01(0.87, 1.17)	0.896	0.94(0.84, 1.06)	0.32
>9 h	0.88(0.53, 1.47)	0.64	0.87(0.43, 1.74)	0.69	0.82(0.38, 1.81)	0.63	1.36(0.86, 2.16)	0.189	1.12(0.76, 1.64)	0.58
**Physical activity**										
Insufficient	1		1		1		1		1	
General	0.99(0.91, 1.06)	0.73	0.98(0.88, 1.09)	0.67	0.99(0.88, 1.11)	0.805	0.95(0.88, 1.03)	0.186	0.99(0.93, 1.05)	0.75
Sufficient	0.79(0.73, 0.85)	<0.001	0.97(0.88, 1.08)	0.58	0.95(0.85, 1.06)	0.346	0.79(0.73, 0.85)	<0.001	0.81(0.76, 0.86)	<0.001
**Smoking status**										
Never smoker	1		1		1		1		1	
Ever smoker	1.11(0.97, 1.26)	0.12	1.01(0.83, 1.24)	0.89	0.83(0.66, 1.05)	0.115	1.01(0.88, 1.16)	0.9	1.04(0.93, 1.16)	0.53
Current smoker	1.64(1.52, 1.76)	<0.001	1.11(0.99, 1.25)	0.07	1.01(0.89, 1.14)	0.895	1.54(1.43, 1.66)	<0.001	1.54(1.45, 1.64)	<0.001
**Drinking status**										
Never drinker	1		1		1		1		1	
Ever drinker	0.99(0.79, 1.28)	0.97	0.42(0.25, 0.73)	0	0.52(0.30, 0.88)	0.015	1.12(0.88, 1.42)	0.37	1.02(0.82, 1.26)	0.89
Current drinker	1.24(1.15, 1.33)	<0.001	1.06(0.95, 1.18)	0.29	0.93(0.83, 1.04)	0.207	0.75(0.70, 0.81)	<0.001	0.96(0.91, 1.02)	0.23
**CHD family history**										
No	1		1		1		1		1	
Yes	0.99(0.85, 1.15)	0.97	1.10(0.90, 1.34)	0.34	1.13(0.91, 1.40)	0.262	1.06(0.91, 1.24)	0.424	1.03(0.91, 1.16)	0.67
**CHD**										
No	1		1		1		1		1	
Yes	0.50(0.34, 0.73)	<0.001	0.28(0.13, 0.64)	0	0.42(0.20, 0.89)	0.024	1.12(0.81, 1.55)	0.475	0.69(0.51, 0.92)	0.01
**Hypertension**										
No	1		1		1		1		1	
Yes	1.67(1.55, 1.80)	<0.001	1.00(0.90, 1.13)	0.94	0.92(0.81, 1.04)	0.188	1.31(1.21, 1.42)	<0.001	1.43(1.34, 1.53)	<0.001
**Diabetes**										
No	1		1		1		1		1	
Yes	1.81(1.63, 2.01)	<0.001	1.36(1.16, 1.59)	<0.001	1.25(1.05, 1.49)	0.012	1.74(1.56, 1.94)	<0.001	1.81(1.63, 1.99)	<0.001
**Obesity**										
No	1		1		1		1		1	
Yes	2.19(2.03, 2.36)	<0.001	1.30(1.15, 1.46)	<0.001	1.39(1.22, 1.57)	<0.001	2.21(2.04, 2.38)	<0.001	2.33(2.18, 2.49)	<0.001

## Discussion

Dyslipidemia is a major modifiable risk factor for CVD, which is a leading cause of morbidity and mortality worldwide [35, 36]. Therefore, it is essential to understand the epidemiological characteristics and risk factors of dyslipidemia in different populations and to implement targeted preventive measures. This study is one of the largest research projects that comprehensively assessed the prevalence of dyslipidemia and its associated factors among sedentary occupational population characterized by low physical activity and high mental workload.

We found that the prevalence of dyslipidemia in our study population was 29.10%, which was lower than the national average of 40.4% and also lower than the results reported in previous studies conducted in other regions of China, such as Beijing, Inner Mongolia, Shenzhen, Shandong, and Guizhou [9, 29, 37–39]. One possible explanation for this finding is that our study population was mainly from the Shanghai area, which is one of the first pilot cities for the Healthy City initiative in China. Shanghai has implemented various health-related policies and conducted large-scale health education activities in all communities every year to enhance residents’ self-management capabilities in health [40–42]. As a result, the health literacy level of Shanghai citizens is higher than the national average. Moreover, our study population had a high level of education, with 93% having a bachelor’s degree or above, and a strong awareness of health. These factors may have influenced their health behaviors, such as dietary habits, physical activity, smoking, and alcohol consumption, which in turn affect their lipid profiles. It could mean that improving health literacy and promoting healthy behaviors are important strategies for preventing and controlling dyslipidemia among occupational populations in China.

We found that the most common abnormal component of dyslipidemia was high triglycerides (TG), which is consistent with previous research findings [20, 35, 43]. The mean levels of TC, TG, HDL-C, and LDL-C in our study were 4.83 mmol/L, 1.51 mmol/L, 1.36 mmol/L, and 2.97 mmol/L, respectively. These levels were higher than those reported by the 2012 Chinese National Nutrition and Health Survey (CNHS), which may attribute to the characteristic of light physical work of occupational population studied in this research [37].

We also observed that the prevalence of dyslipidemia and its components increased with age, especially in the 30–49 age group, in line with earlier studies [35, 44, 45]. This age group is a critical period for controlling blood lipids to prevent cardiovascular diseases, as blood lipid levels tend to rise rapidly during this period [46]. Moreover, we found that male and married status were risk factors for dyslipidemia, high TG, and low HDL-C. Although the reason has not been proven, it is possible that men have a higher prevalence of unhealthy lifestyle such as insufficient vegetable intake, late-night diet, high intake of animal offal or meat, smoking and alcohol consumption, combined with the results of the sample population with dyslipidemia. Furthermore, we noted that hypertension, diabetes, and smoking were risk factors for dyslipidemia, while a history of CHD was a protective factor for dyslipidemia and its components (excluding low HDL-C). This may be explained by the fact that CHD patients are more likely to be aware of their lipid abnormalities and receive treatment and control for them [47].

Our findings revealed a positive association between drinking and TG levels, which is in line with previous studies [48–50]. However, this association was not significant after adjusting for other variables in a multivariate regression analysis. This may be due to the complex effects of alcohol intake on blood lipid levels. Previous research has suggested that moderate drinking may have some health benefits, such as reducing the risk of cardiovascular disease and dementia [51, 52]. However, these benefits are not linear, and excessive drinking may increase the risk of various health problems. Therefore, the lack of a clear association between drinking and dyslipidemia in our study may reflect the balance or cancellation of the protective or harmful effects of different levels of alcohol consumption in our sample population. Further studies are needed to explore the optimal range and pattern of drinking for preventing dyslipidemia.

Physical activity is widely recognized as a key factor for maintaining good health, while sedentary behavior is associated with an increased risk of several chronic diseases (such as cardiovascular disease and cerebrovascular events) [53, 54]. Sedentary behavior has become a major public health concern in modern society. For adults, sedentary time could be divided into workplace sedentary time and leisure sedentary time [15]. Our study examined the association between leisure sedentary time and dyslipidemia among sedentary occupational population, and found that leisure sedentary time was a factor associated with dyslipidemia and all its components. We suggest that interventions to reduce sedentary behavior should be tailored to the specific needs and characteristics of different occupational groups, considering their age, social role, and life role. For example, office workers may benefit from regular breaks and physical activities during their own time [55].

Our study has some potential limitations that should be acknowledged. First, this was a single-center study, which may limit the generalizability of our findings to other sedentary occupational populations. However, the large sample size in our study may partly compensate for this limitation. Second, our study relied on self-reported information and recalled data, which may introduce sources of bias such as recall bias and measurement bias. Therefore, the accuracy and validity of the data may be affected by these factors. Third, our study used a cross-sectional design, which precludes the establishment of causal relationships between dyslipidemia and its risk factors. Longitudinal studies are needed to confirm the direction and magnitude of these associations. Fourth, our study did not consider the use of hypolipidemic drugs that might reduce the evidence sources for explaining the prevalence of dyslipidemia.

## Conclusions

Our study provides valuable and current epidemiological data on dyslipidemia and its potential risk factors among sedentary occupational populations in China. The prevalence of dyslipidemia among sedentary occupational population in our study was lower than in other previous studies without the limitation of occupational characteristics. Dyslipidemia among sedentary occupational population is associated with several modifiable factors such as BMI, smoking, drinking, physical activity, and dietary habits. These findings could inform the development of effective strategies for the prevention and control of dyslipidemia in sedentary occupational populations, such as health education, lifestyle modification, and regular screening.

## Data Availability

The data that support this study are not publicly available due to participants’ privacy protection but can be obtained from the corresponding author upon reasonable request. Researchers who are interested in our study can contact the corresponding author Dr. Tiantian Zhang (tiantianzhang18@fudan.edu.cn) who will review the data request.

## Abbreviations

CVD: Cardiovascular disease
TC: Total cholesterol
LDL-C: low-density lipoprotein cholesterol
TG: Triglycerides
HDL-C: High-density lipoprotein cholesterol
CHD: Coronary heart disease
FBG: Fasting blood glucose
BMI: Body mass index
WC: Waist circumference
SBP: Systolic blood pressure
DBP: Diastolic blood pressure
SD: Standard deviation
ORs: Odds ratios
CNHS: Chinese National Nutrition and Health Survey
